# Subdural Thoracolumbar Spine Hematoma after Spinal Anesthesia: A Rare Occurrence and Literature Review of Spinal Hematomas after Spinal Anesthesia

**DOI:** 10.7759/cureus.1032

**Published:** 2017-02-16

**Authors:** Prasanthi Maddali, Blake Walker, Christian Fisahn, Jeni Page, Vicki Diaz, Michael E Zwillman, Rod J Oskouian, R. Shane Tubbs, Marc Moisi

**Affiliations:** 1 Neurosurgery, Seattle Science Foundation; 2 Neurological Surgery, Wayne State University; 3 Orthopedic Surgery, Swedish Neuroscience Institute; 4 Neurosurgery, Swedish Neuroscience Institute; 5 Anesthesiology and Critical Care, Houston Methodist Hospital; 6 Neurosurgery, Complex Spine, Swedish Neuroscience Institute; 7 Seattle Science Foundation

**Keywords:** spinal epidural hematoma, spinal subdural hematoma, spinal anesthesia, spinal epidural anesthesia

## Abstract

Spinal hematomas are a rare but serious complication of spinal epidural anesthesia and are typically seen in the epidural space; however, they have been documented in the subdural space. Spinal subdural hematomas likely exist within a traumatically induced space within the dural border cell layer, rather than an anatomical subdural space. Spinal subdural hematomas present a dangerous clinical situation as they have the potential to cause significant compression of neural elements and can be easily mistaken for spinal epidural hematomas. Ultrasound can be an effective modality to diagnose subdural hematoma when no epidural blood is visualized. We have reviewed the literature and present a full literature review and a case presentation of an 82-year-old male who developed a thoracolumbar spinal subdural hematoma after spinal epidural anesthesia. Anticoagulant therapy is an important predisposing risk factor for spinal epidural hematomas and likely also predispose to spinal subdural hematomas. It is important to consider spinal subdural hematomas in addition to spinal epidural hematomas in patients who develop weakness after spinal epidural anesthesia, especially in patients who have received anticoagulation.

## Introduction and background

Spinal canal hematomas have long been recognized as lesions capable of producing sudden spinal cord and/or cauda equina compression [1-19]. Different factors play an important role including ruptured vascular malformations, underlying neoplasm, hypertension, coagulopathies, trauma, pregnancy, old age, infection, and spinal surgery [8, 13-14, 20-22]. It is critical for this to be in the postoperative differential diagnosis of patients who have undergone spinal anesthesia, especially if on anticoagulation therapy.

We present a representative case of an 82-year-old man who underwent a failed attempt at spinal anesthesia for left iliofemoral endarterectomy with patch angioplasty and bilateral common iliac artery iCAST stents (Atrium Medical, Hudson, NH, US) and developed a thoracolumbar subdural hematoma while on anticoagulation postoperatively.

informed consent was obtained from the patient for this study.

### Representative case

**Figure 1:**
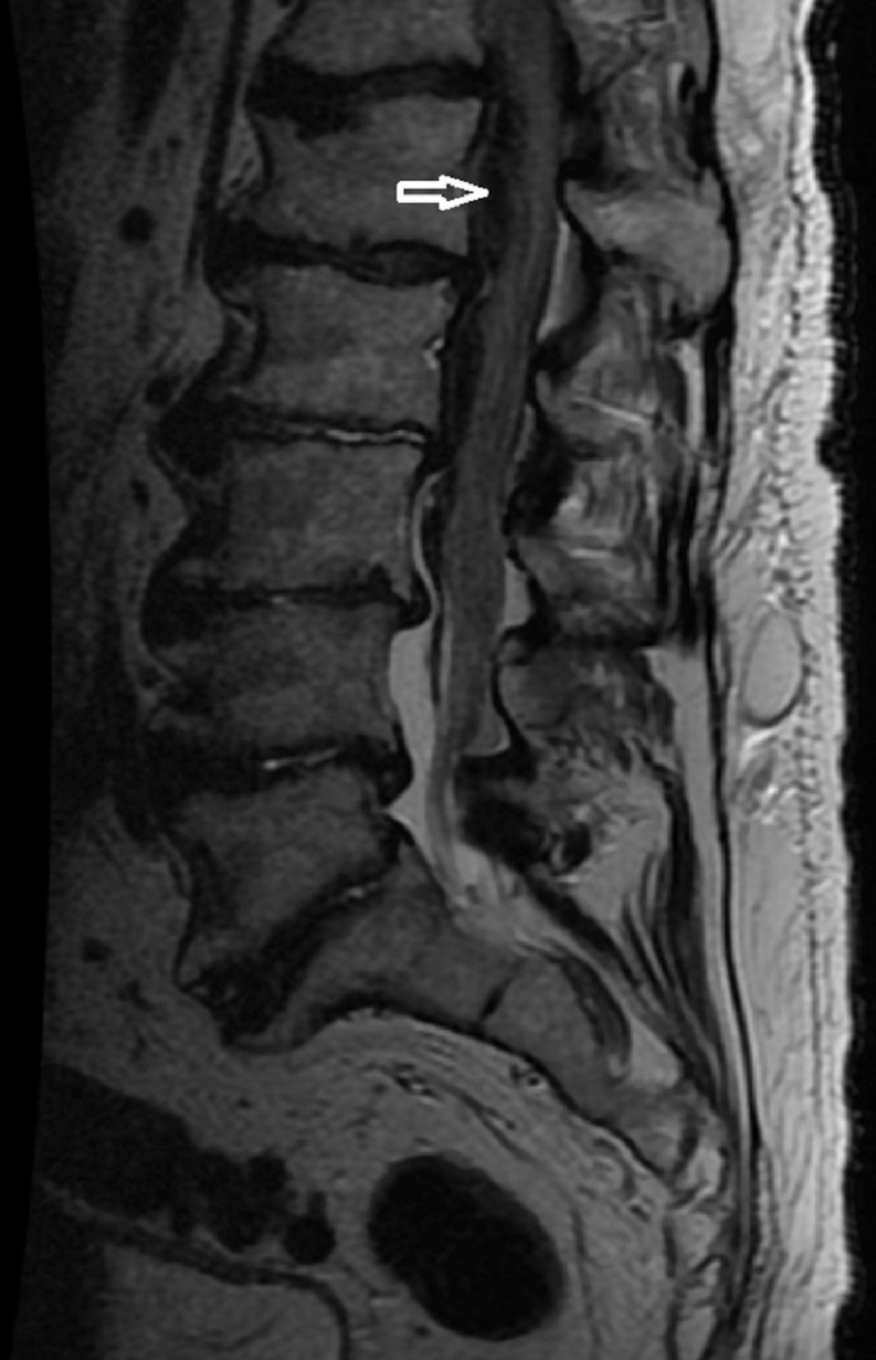
MRI Lumbar Spine T2 Weighted White arrow pointing to hematoma.

The patient is an 82-year-old male with a history of hypertension and peripheral vascular disease with claudication, who underwent a left iliofemoral endarterectomy with patch angioplasty and bilateral common iliac artery iCAST stents. Spinal anesthesia was attempted but failed, and he ultimately required general anesthesia. The surgical procedure was uneventful. In the postoperative period, he was treated with aspirin (81 mg) and Plavix (75 mg), as well as Lovenox for deep vein thrombosis prophylaxis. On postoperative day 1 (POD), he noticed weakness in his bilateral lower extremities while ambulating. On POD 3, he noticed a dense paraparesis, and on POD 4, he was paraplegic. An MRI scan obtained of the thoracic and lumbar spine showed a large epidural hematoma with central canal stenosis extending most prominently from T10-T11 to L1 (Figure 1). 

**Figure 2:**
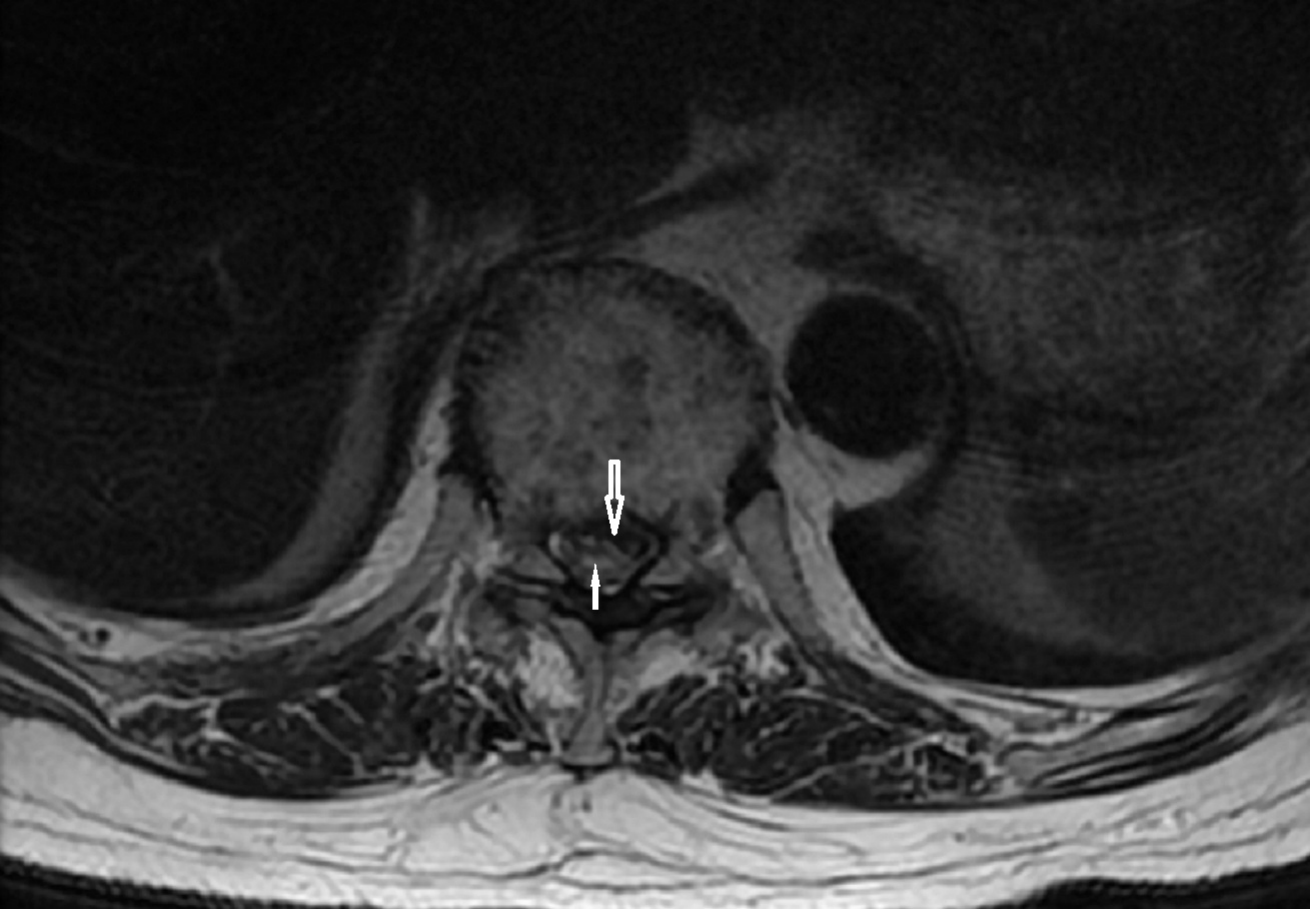
MRI Axial Thoracic Spine T2 Weighted Unfilled arrow shows hematoma. Filled arrow shows edema in the thoracic spinal cord.

**Figure 3:**
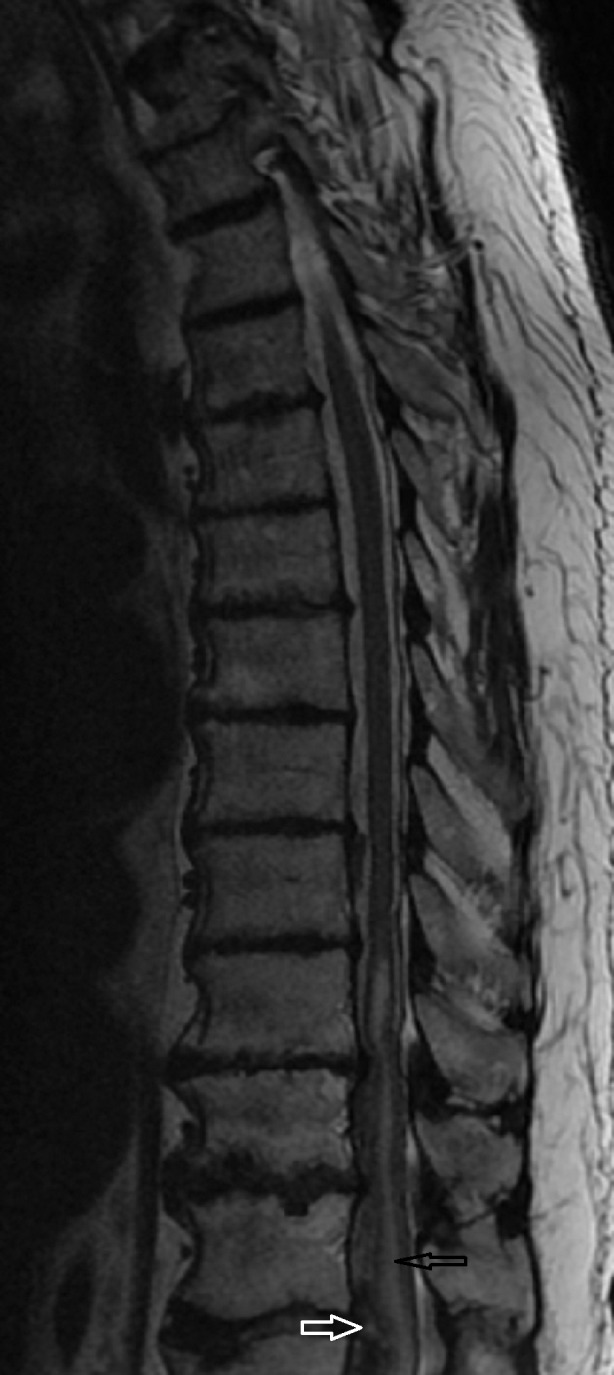
MRI Thoracic Spine T2 Weighted Black arrow shows hematoma. White arrow shows edema in the thoracic spinal cord.

Edema within the cord extends from T10 to the conus as shown in Figures 2-3. 

He was emergently transferred to our hospital, and on neurological exam, he was found to have a T10 sensory level and motor exam consistent with 0-1/5 in his right lower extremity and 0/5 in his left lower extremity. He was therefore immediately taken to the operative theater for decompression and evacuation of the hematoma. He underwent a T9-L1 laminectomy; however, when the decompression was completed, we were unable to identify any epidural hematoma. The dura was exceptionally tense, and therefore, an ultrasound was performed that demonstrated that the entire hematoma was intradural. The dura was opened the entire length of the laminectomy and an acute hematoma under high pressure erupted. The hematoma was irrigated out with good decompression of the neural elements, and the dura was completely relaxed after closure. Unfortunately, postoperatively, the patient never regained lower extremity motor function and went to a skilled nursing facility. 

## Review

Anticoagulant therapy, especially in combination with spinal punctures or epidural anesthesia, has been regarded as an important predisposing factor for spinal epidural hematoma [2, 6, 14, 23-25]. In fact, anticoagulation initiated within one hour after a traumatic lumbar puncture and clotting studies greater than two times normal have been reported to increase the risk of bleeding [14, 23-25]. Spinal hematomas, however, have also been described as occurring spontaneously or after only minor activities such as sneezing or coughing [13, 20-22, 26-29]. Although usually acute (occurring within the first 48 hours of the event), some spinal hematomas have been identified as causes of chronic myelopathy [7, 21-22, 29, 30]. Because they rarely spontaneously remit, and because of their great propensity for causing severe irreversible neurologic deficits, immediate surgical evacuation of these compressive lesions is strongly advocated [1, 9, 13, 15, 17].

Most commonly, spinal hematoma is seen in the epidural space and rarely seen in the subdural space or subarachnoid space; these hematomas can cause neural compression comparable or worse to that of an epidural hematoma [1-8, 24, 28, 31-32]. The correct preoperative diagnostic location of the hematoma informs the surgeon of the need to open the dura or arachnoid to locate the hematoma, particularly in cases complicated by the coexistence of epidural and subdural hematomas. As we learned from our case, it is the better part of valor to always check with an ultrasound to confirm that there is no subdural component of the hematoma, especially when an epidural hematoma is not visualized.

The mechanism of hematoma formation in the spinal subdural space remains open to conjecture. However, an electron microscopy study by Haines, et al. recently offered explanations for subdural hematoma occurrence in the brain, which also seem plausible for the spine [33].  According to these authors, the dura is composed externally of elongated flattened fibroblasts and large amounts of extracellular collagen; internally, it is made up of flattened fibroblasts and extracellular spaces containing no extracellular collagen, with few cell junctions. While the external layer of dura is strong, the inner dura or meningeal dura, also known as the dural border cell layer, is structurally weak and vulnerable to tearing open from injury. The adjacent arachnoid layer bears resemblance to the external dura in that it is also a strong barrier, being composed of larger cells with many cell junctions, no extracellular collagen, and no extracellular space. The numerous tight junctions in this layer prevent the free movement of fluids and ions. This arachnoid layer is further strengthened by the fact that arachnoid trabeculae formed from specialized fibroblasts attached to the inner arachnoid layer traverse the subarachnoid space and attach to the pia. Under these anatomic conditions, no potential space exists at the dura-arachnoid junction in the brain. Rather, the dural border cell layer, which is the structurally weakest point in the meninges, cleaves open when traumatic or other pathologic forces cause tissue injury. The space that is created in this fashion is actually within this distinct dural border cell layer as opposed to being in an anatomic subdural space. Based on these anatomical observations, spinal hematomas can be classified as inner dural or true subdural type. According to Haines, et al., it is not difficult to imagine that in patients with preexisting coagulopathy, this layer is more prone to rupture especially after the trauma of an attempted spinal anesthesia has occurred [33].

## Conclusions

Thoracolumbar spinal canal hematomas have long been known as lesions that are capable of producing sudden spinal cord and/or cauda equina compression. It is important to consider spinal subdural hematoma in patients undergoing spinal anesthesia who develop neurological deficits, especially when there is anticoagulation on board. When evacuating the hematoma, it is critical to check both the epidural and subdural space using an ultrasound. 

## References

[1] Kirkpatrick D, Goodman SJ (1975). Combined subarachnoid and subdural spinal hematoma following spinal puncture. Surg Neurol.

[2] Edelson RN, Chernik NL, Posner JB (1974). Spinal subdural hematomas complicating lumbar puncture: occurrence in thrombocytopenic patients. Arch Neurol.

[3] Schiller F, Neligan G, Budtz-Olsen 0 (1948). Surgery in haemophilia: a case of spinal subdural hematoma producing paraplegia. Lancet.

[4] Wolcott GJ, Grunnet ML, Lahey ME (1970). Spinal subdural hematoma in a leukemic child. J Pediatr.

[5] Schaake T, Schafer ER (1970). Spontaneous hemorrhage in the spinal canal. J Neurol Neurosurg Psychiatry.

[6] Anagnostopoulos Dl, Gortvai P (1972). Spontaneous spinal subdural hematoma. Br Med J.

[7] Stewart DH, Watkins ES (1969). Spinal cord compression by chronic subdural hematoma. J Neurosurg.

[8] Ainslie JP (1958). Paraplegia due to spontaneous extradural or subdural hemorrhage. Br J Surg.

[9] Packer NP, Cummins BH (1978). Spontaneous epidural hemorrhage: a surgical emergency. Lancet.

[10] DeAngelis J (1972). Hazards of subdural and epidural anesthesia during anticoagulant therapy: a case report and review. Anesth Analg.

[11] Dawson BH (1963). Paraplegia due to spinal epidural hematoma. J Neurol Neurosurg Psychiatry.

[12] Cooper DW (1967). Spontaneous spinal epidural hematoma. J Neurosurg.

[13] Pear BL (1972). Spinal epidural hematoma. Am J Roentgenol Radium Ther Nucl Med.

[14] Alderman DB (1956). Extradural spinal cord hematoma: report of a case due to dicumarol and review of the literature. N Engl J Med.

[15] Beatty RM, Winston KR (1984). Spontaneous cervical epidural hematoma: a consideration of etiology. J Neurosurg.

[16] Lowrey JJ (1959). Spinal epidural hematomas: experiences with three patients. J Neurosurg.

[17] Grollmus J, Hoff J (1975). Spontaneous epidural haemorrhage: good results after early treatment. J Neural Neurosurg Psychiatry.

[18] Markham JW, Lynge HN, Stahlman GEB (1967). The syndrome of spontaneous spinal epidural hematoma. J Neurosurg.

[19] Harris ME (1969). Spontaneous epidural spinal hemorrhage. AJR Am J Roentgenol.

[20] Harik Sl, Raichle ME, Reis DJ (2017). Spontaneously remitting spinal epidural hematoma in a patient on anticoagulants. N Engl J Med.

[21] Boyd HR, Pear BL (1972). Chronic spontaneous spinal epidural hematoma: report of two cases. J Neurosurg.

[22] Hehman K, Norrell H (1968). Massive chronic spinal epidural hematoma in a child. Am J Dis Child.

[23] Senelick RC, Norwood CW, Cohen GH (1976). "Painless" spinal epidural hematoma during anticoagulant therapy. Neurology.

[24] Brem SS, Hafler DA, Van Llitert RL, Ruff RL, Reichert WH (1981). Spinal subarachnoid hematoma: a hazard of lumbar puncture resulting in reversible paraplegia. N Engl J Med.

[25] Ruff RL, Dougherty JH Jr (1981). Evaluation of acute cerebral ischemia for anticoagulant therapy: computed tomography or lumbar puncture. Neurology.

[26] Gold ME (1963). Spontaneous spinal epidural hematoma. Radiology.

[27] Tsai FY, Popp AJ, Waldman J (1975). Spontaneous spinal epidural hematoma. Neuroradiology.

[28] Slavin HB (1937). Spontaneous intraspinal subarachnoid hemorrhage: report of a case. J Nerv Ment Dis.

[29] Plotkin R, Ronthal M, Froman C (1966). Spontaneous spinal subarachnoid hemorrhage: report of 3 cases. J Neurosurg.

[30] Levitan LH, Wiens CW (1983). Chronic lumbar extradural hematoma: CT findings. Radiology.

[31] Masdeu JC, Breuer AC, Schoene WC (1979). Spinal subarachnoid hematomas clue to a source of bleeding in traumatic lumbar puncture. Neurology.

[32] Rengachary SS, Murphy D (1974). Subarachnoid hematoma following lumbar puncture causing compression of the cauda equina. J Neurosurg.

[33] Haines DE, Harkey HL, AI-Mefty 0 (20). The "subdural" space: a new look at an outdated concept. Neurosurgery.

